# Co-expression with the Type 3 Secretion Chaperone CesT from Enterohemorrhagic *E. coli* Increases Accumulation of Recombinant Tir in Plant Chloroplasts

**DOI:** 10.3389/fpls.2017.00283

**Published:** 2017-03-06

**Authors:** Jacqueline MacDonald, Sean Miletic, Typhanie Gaildry, Adam Chin-Fatt, Rima Menassa

**Affiliations:** ^1^London Research and Development Centre, Agriculture and Agri-Food Canada, LondonON, Canada; ^2^Department of Biology, University of Western Ontario, LondonON, Canada; ^3^Department of Biology, Université de BordeauxTalence, France

**Keywords:** chloroplast targeting, EHEC, EPEC, fluorescence microscopy, T3SS, translocated intimin receptor, transplastomic, subunit vaccine

## Abstract

Type 3 secretion systems (T3SSs) are utilized by pathogenic *Escherichia coli* to infect their hosts and many proteins from these systems are affected by chaperones specific to T3SS-containing bacteria. Toward developing a recombinant vaccine against enterohaemorrhagic *E. coli* (EHEC), we expressed recombinant T3SS and related proteins from predominant EHEC serotypes in *Nicotiana* chloroplasts. *Nicotiana benthamiana* were transiently transformed to express chloroplast-targeted Tir, NleA, and EspD from the EHEC serotype O157:H7; a fusion of EspA proteins from serotypes O157:H7 and O26:H11; and a fusion of epitopes of Tir (Tir-ep) from serotypes O157:H7, O26:H11, O45:H2, and O111:H8. C-terminal GFP reporter fusion constructs were also developed and transiently expressed to confirm subcellular localization and quantify relative expression levels *in situ*. Recombinant proteins were co-expressed with chaperones specific to each T3SS protein with the goal of increasing their accumulation in the chloroplast. We found that co-expression with the chloroplast-targeted chaperone CesT significantly increases accumulation of recombinant Tir when the latter is either transiently expressed in the nucleus and targeted to the chloroplast of *N. benthamiana* or stably expressed in transplastomic *Nicotiana tabacum*. CesT also helped maintain higher levels of Tir:GFP fusion protein over time both *in vivo* and *ex vivo*, indicating that the favorable effect of CesT on accumulation of Tir is not specific to a single time point or to fresh material. By contrast, T3SS chaperones CesT, CesAB, CesD, and CesD2 did not increase accumulation of NleA:GFP, EspA:GFP, or EspD:GFP, which suggests dissimilar functioning of these chaperone–substrate combinations. CesT did not increase accumulation of Tir-ep:GFP, which may be due to the absence of the CesT binding domain from this fusion protein. The fusion to GFP improved accumulation of Tir-ep relative to the unfused protein, but not for the other recombinant proteins. These results emphasize the importance of native chaperones and stabilizing fusions as potential tools for the production of higher levels of recombinant proteins in plants; and may have implications for understanding interactions between T3SS chaperones and their substrates. In particular, our findings highlight the potential of T3SS chaperones to increase accumulation of recombinant T3SS proteins in heterologous systems.

## Introduction

*Escherichia coli* is a Gram-negative bacterium, which is a ubiquitous commensal resident of the intestinal microflora of warm blooded animals, including humans. However, several *E. coli* strains cause disease in animals and/or humans. Among these are EHEC and EPEC, which utilize a bacterial T3SS to infect their hosts.

The T3SS, a common feature of many pathogenic bacteria, is a needle-like structure used to transport bacterial proteins directly into the host cell. It is encoded from a single pathogenicity island on the bacterial chromosome, known as the LEE. The T3SS is composed of a basal body than spans the bacterial inner and outer membranes, associated with an extracellular needle (or pilus in bacterial pathogens of plants; Buttner, 2012). In EHEC and EPEC, an extension of the needle is provided by a filament composed of subunits of EspA (Daniell et al., 2001). This filament connects the needle to a translocation pore within the plasma membrane of the host cell, which is composed of the bacterial proteins EspB and EspD (Buttner, 2012). In addition to its role in protein secretion, the filament is involved in adhesion to host cells (Cleary et al., 2004) and to leaf epidermis, whereby leaves act as an intermediary for indirect transmission between animals (Shaw et al., 2008).

Once the T3SS apparatus is formed, bacterial proteins can be secreted through the T3SS and into the cytosol of the host cell. In EHEC and EPEC, the first such effector protein is Tir (Buttner, 2012). Now inside the host cell, Tir integrates into the plasma membrane where it serves as a receptor for the bacterial outer membrane protein, Intimin, enabling intimate attachment of the bacterium to the host (Kenny et al., 1997). Another effector protein, NleA, localizes to the Golgi apparatus (Gruenheid et al., 2004) and is believed to disrupt intestinal tight junctions and inhibit protein trafficking in the host cells (Kim et al., 2007; Thanabalasuriar et al., 2010).

Type 3 secretion system and associated effector proteins have been produced recombinantly in bacteria and eukaryotes in order to study their structure or as candidates for vaccine research. In some cases, however, the recombinant protein yields have been abnormally low (Dasanayake et al., 2011; *Miletic in preparation*); possibly because many T3SS proteins are stabilized or otherwise affected by specific chaperone proteins that are present in pathogenic *E. coli* (Burkinshaw and Strynadka, 2014). Several chaperones are encoded on the LEE, and mutations of such chaperones in EHEC or EPEC result in decreased secretion and decreased accumulation of EspA, EspD, Tir, or NleA, but do not seem to affect their mRNA levels (**Table 1**; Wainwright and Kaper, 1998; Elliott et al., 1999; Creasey et al., 2003; Neves et al., 2003; Su et al., 2008; Ku et al., 2009; Ramu et al., 2013). Each type 3 secretion chaperone targets a small number of substrate proteins and may function to prevent premature aggregation or to direct secretion through the T3SS (Francis, 2010).

**Table 1:** Effects of chaperone loss-of-function mutations on protein accumulation in pathogenic *Escherichia coli.*

Chaperone	Protein of Interest				Reference
	EspA	EspD	Tir	NleA	
CesAB	✓	✖	✖	–	Creasey et al., 2003
CesD	✓	✓	–	–	Wainwright and Kaper, 1998
CesD2	✖	✓	–	–	Neves et al., 2003
CesT	✖	✖	✓	✓	Elliott et al., 1999; Thomas et al., 2005; Ramu et al., 2013

*✓Mutation of chaperone results in lowered accumulation in *E. coli*. ✖Chaperone mutation does not affect accumulation. – Effect not tested.*

We transformed plants with constructs to express components of the T3SS and effector proteins in the chloroplasts, as candidates for vaccine research. Chloroplast-targeted, nuclear-encoded, transient expression in *N. benthamiana* was initially used as a model for transplastomic expression in *N. tabacum*. As chloroplast DNA is rarely transmitted through pollen (Ruf et al., 2007; Svab and Maliga, 2007) chloroplast transformation of male-sterile lines of *N. tabacum*, such as the one used in this study, virtually eliminates concerns of transgene escape via pollen; a favorable safety feature for scale-up of plant-made vaccine antigens.

As T3SS chaperones are not encoded in the chloroplast or nuclear genomes of plants, we hypothesized that co-expression with appropriate T3SS chaperones would increase accumulation of the recombinant EHEC proteins in *Nicotiana* chloroplasts, possibly through stabilization. To this end, each T3SS transgene was fused at the 3′ end with a green fluorescent protein (GFP) reporter gene and transiently expressed in *N. benthamiana* in order to confirm subcellular localization and quantify relative expression levels *in situ*. The effects of chloroplast-targeted T3SS chaperones on recombinant protein accumulation were determined by comparing relative fluorescent signals from GFP fusions with and without co-expressed T3SS chaperones. We confirmed a positive effect of the T3SS chaperone CesT on accumulation of Tir by comparing Western blot signals from transiently expressed chloroplast-targeted Tir:GFP and Tir with and without co-expression of CesT, and from transplastomically expressed Tir with and without chloroplast-targeted CesT.

## Materials and Methods

### Expression Constructs and Transformations

Genes for chloroplast-targeted expression encoded the T3SS proteins EspA and EspD, the T3SS effector proteins Tir and NleA, and the chaperones CesT, CesAB, CesD, and CesD2. The EspA construct (EspA) contained a fusion of EspA coding regions from two different EHEC serotypes (O157:H7 and O26:H11; GenBank accession numbers WP_000381516.1 and WP_000381555.1, respectively) connected by a GSGGSG linker. The EspD, Tir, and NleA were from EHEC serotype O157:H7 (GenBank accession numbers WP_000935759.1, WP_001301454.1, and WP_001025672.1, respectively). A fusion of epitopes of Tir (Tir-ep) contained the coding regions of the Intimin-binding domains from EHEC serotypes O157:H7 (amino acids 226–405), O111:H8 (amino acids 229–403; the included region has the same sequence as serotype O121:H19), O26:H11 (amino acids 218–390), and O45:H2 (amino acids 218–390) (GenBank accession numbers WP_032310298.1, EHW02833.1, WP_074466017.1, and WP_053879149.1, respectively), connected with GSGGSG linkers. GenBank accession numbers for chaperone proteins are: CesT (WP_000098793.1), CesAB (WP_000029559.1), CesD (WP_000087467.1), CesD2 (WP_000228583.1).

**FIGURE 1:**
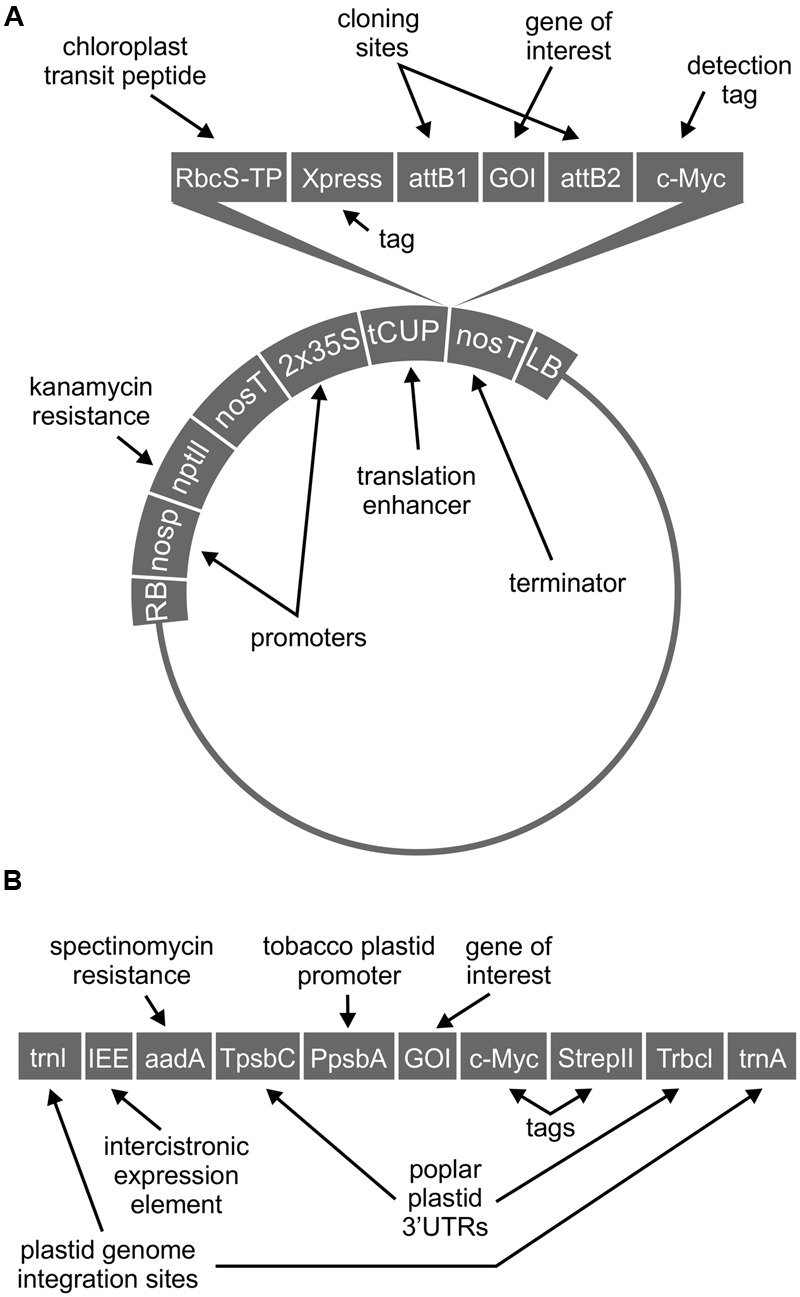
**Schematic representation of cassettes for chloroplast-targeted expression (A)** and for transplastomic expression **(B)**. Schematics not drawn to scale.

Sequences were codon-optimized for expression in *N. benthamiana*, synthesized (BioBasic Inc., Markham, ON, Canada), and cloned by the Gateway^TM^ method (Thermo Fisher Scientific Inc., Waltham, MA, USA) into an in-house pCaMGate expression vector for chloroplast-targeted expression (Pereira et al., 2014). This vector includes coding regions for an N-terminal chloroplast transit peptide from the ribulose-1,5-bisphosphate carboxylase oxygenase (RuBisCO) small subunit of *N. tabacum*, and a C-terminal c-myc tag for detection (**Figure 1A**). GFP fusions were made by adding the coding region for enhanced GFP to the 3′ end of the T3SS protein coding region, upstream of the Gateway^TM^ recombination *attb2* site and c-myc tag, using an XbaI restriction site. The T3SS protein and GFP are separated by a serine-arginine linker encoded by the XbaI site (See Supplementary Data for full coding sequences).

For transplastomic expression of Tir, the coding region was codon-optimized for expression in *N. tabacum* chloroplast, synthesized, and inserted into the pCEC5 chloroplast expression vector (pCEC4 without T7g10; Kolotilin et al., 2013), using NheI and NotI restriction sites (**Figure 1B**) (See Supplementary Data for full coding sequences). All coding regions were sequenced to confirm identity and proper insertion of genes. Stable plastome transformation of the male-sterile, low-alkaloid *N. tabacum* cultivar 81V9 (Menassa et al., 2001) was done using standard microparticle bombardment of leaf tissue followed by three consecutive rounds of regeneration on selective medium (500 μg/mL spectinomycin), as previously described (Kolotilin et al., 2013). Transient transformation of *N. benthamiana* was done using standard agro-infiltration (Pereira et al., 2014). For expression of one or two genes of interest, each culture of *Agrobacterium tumefaciens* EHA-105 carrying a different construct of interest was diluted to a final optical density at 600 nm (OD_600_) of 0.45 in Gamborg’s solution (3.2 g/L Gamborg’s B5 salts with vitamins, 20 g/L sucrose, 10 mM MES, pH 5.6, 200 μM acetosyringone), and *A. tumefaciens* carrying a construct for expression of the p19 suppressor of gene silencing (Silhavy et al., 2002) was suspended at a final OD_600_ of 0.10. For co-expression of three genes of interest, each culture of *A. tumefaciens* carrying a different construct of interest was diluted to a final OD_600_ of 0.30, with *A. tumefaciens* carrying the construct for p19 at a final OD_600_ of 0.10. Stable transformation of the *N. tabacum* nuclear genome with chloroplast-targeted CesT was done using *A. tumefaciens* and standard methods (Horsch et al., 1985) followed by regeneration on selective medium (100 μg/mL kanamycin).

### Fluorescence Microtiter Plate Spectroscopy

Fluorescence microtiter plate spectroscopy of intact leaf disks was done as previously described (Pasin et al., 2014). Fifty microliters of water were placed in each well of a Costar flat-bottom black opaque 96 well plate (Corning Inc., Corning, NY, USA) and spread to cover the bottom of the well. Single leaf disks taken with a size 3 cork borer (6 mm diameter) were placed with the adaxial side down on top of the water in the wells. Fluorescence detection was performed using a Synergy 2 reader with Gen5 version 1.10 software (BioTek Instruments Inc., Winooski, VT, USA) and the following read parameters: excitation 485/20, emission 516/50, optics position top 50%, and sensitivity 120.

### Fluorescence Microscopy

To determine subcellular localization of GFP fusion proteins, leaf samples were imaged using an IX83 inverted microscope equipped with a Fluoview FV1200 confocal laser scanner and 60 x water immersion objective, with FV10-ASW version 4.2 software (Olympus Corp., Shinjuku, Japan). Samples were excited at 488 nm using a multi-argon laser set at 5% and emission was detected at 500–545 nm for GFP and at 630–690 nm for chlorophyll. Detector sensitivity (HV) was no higher than 500 for GFP acquisition and images were captured at speeds of 20 or 40 μs/pixel (for the untransformed control, HV was set at 500 and speed at 40 μs/pixel).

### Protein Extraction and Western Blot

Pre-weighed leaf samples were frozen at –80°C and homogenized with silica beads (Bio Spec Products Inc., Bartlesville, OK, USA) for 2 min using a TissueLyser II (Retsch Inc., Newton, PA, USA). Five milliliters of extraction buffer I (2 M urea, 4% SDS, 8% sucrose) or extraction buffer II (1x PBS, pH 7.8, 4% SDS, 1 mM EDTA, 2% PVPP, 100 mM sodium ascorbate, 8 M sucrose, 1 μg/mL leupeptin, 1 mM PMSF, 1 μg/mL pepstatin A) were added per gram of sample. For incremental leaf samples (See Results section entitled “Co-expression with CesT Increases Accumulation of Transplastomic Tir”), the extract was sonicated using a Sonic Dismembrator model 100 (Thermo Fisher Scientific Inc., Waltham, MA, USA) on ice. All samples were then vortexed on high speed for 30 s and centrifuged at 20,000 × *g* for 10 min. Liquid protein samples were combined with 5x loading buffer (0.3 M Tris-HCl pH 8.0, 5% SDS, 10% glycerol, 100 mM DTT, 0.05% Phenol Red), heated at 99°C for 5 min, then loaded onto Express Plus PAGE 4-20% gels (Genscript Inc., Piscataway, NJ, USA). Gels were run at 100 V for 100–110 min, then transferred to polyvinylidine difluoride (PVDF) membrane using the Trans-Blot Turbo transfer system (Bio-Rad Laboratories Inc., Hercules, CA, USA). Blots were blocked with 5% skimmed milk in *tris*-buffered saline, pH 7.5, and proteins of interest were probed with anti-c-myc antibody from mouse (Genscript Inc., Piscataway, NJ, USA) and the One-Hour Basic Western kit for mouse primary antibody (Genscript Inc., Piscataway, NJ, USA). Detection was performed using Amersham ECL Western Blot detection reagents (GE Healthcare, Mississauga, ON, Canada) or Enhanced Chemiluminescent detection solution (Biorad Laboratories Inc., Hercules, CA, USA) and a MicroChemi 4.2 imaging system with GelCapture acquisition software (DNA Bio-Imaging Systems Ltd., Jerusalem, Israel). For staining, membranes were rinsed in methanol followed by ultrapure water, stained using GelCode Blue (Thermo Fisher Scientific Inc., Waltham, MA, USA) for 15 min, and destained in 50% methanol 1% acetic acid for 15 min.

### Calculations

Paired *t*-tests were done using GraphPad QuickCalcs (GraphPad Software Inc., La Jolla, CA, USA). Protein sizes were predicted based on amino acid sequence only, using an online peptide calculator tool (Genscript Inc.; https://www.genscript.com/ssl-bin/peptide_mw).

## Results

### Relative Quantities of GFP Fusion Proteins Can Be Estimated from Intact Leaf Disks

The T3SS transgenes were fused to the *gfp* reporter gene in order to visualize and quantify relative protein accumulation levels by microscopy *in situ*, as well as by Western blot. Direct observation *in situ* can provide a more realistic idea of a recombinant protein’s accumulation since membrane proteins have a tendency to resist solubilization during extraction for Western blot detection. Several of the proteins of interest can associate with membranes. Following agro-infiltration of *N. benthamiana* plants, relative amounts of the transiently expressed fusion proteins were determined by measuring GFP signals with fluorescence microtiter plate spectroscopy of intact leaf disks, as has been done previously (Pasin et al., 2014). These relative measurements correlated well with visual estimates of GFP signals from fluorescence microscopy of the same leaf disks, supporting the use of leaf disk spectroscopy in subsequent analyses (Supplementary Figure S1).

### GFP Fusions Affect Recombinant Protein Accumulation

Similar to our previous Western blot results where recombinant EspD was not detected (Miletic et al. *unpublished*), fluorescence spectroscopy and microscopy revealed little to no accumulation of EspD:GFP (Supplementary Figure S1). However, while Tir-ep was not previously detected by Western blot, Tir-ep:GFP did produce a fluorescence signal above that of untransformed WT leaves, which served as a negative control. To determine effects of the GFP fusion on accumulation of the recombinant EHEC proteins, accumulation of the GFP fusions was compared with that of the unfused EHEC proteins via Western blot.

**FIGURE 2:**
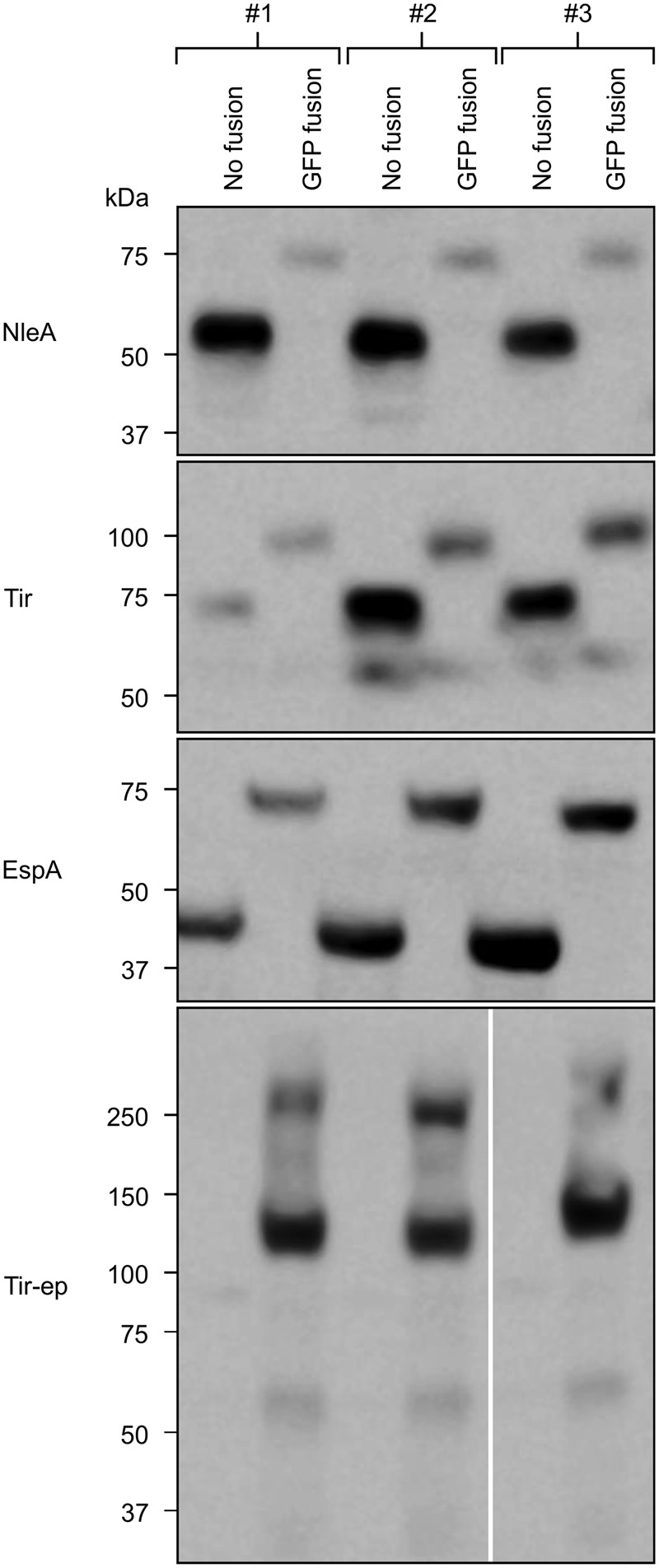
**GFP fusions affect recombinant protein accumulation.** Recombinant proteins were targeted to the plant chloroplasts using the transit peptide from the small subunit of tobacco RuBisCO, and expressed transiently in agro-infiltrated *N. benthamiana* along with the p19 suppressor of gene silencing. On each of three plants (#1–3), a single leaf was infiltrated with an unfused EHEC protein and its corresponding GFP fusion on opposite sides of the mid-vein. Infiltrated plant tissue was harvested 4 dpi. Predicted protein sizes, based on amino acid composition, are: NleA, 58 kDa; NleA:GFP, 85 kDa; EspA, 51 kDa; EspA:GFP, 79 kDa; Tir, 68 kDa; Tir:GFP, 95 kDa; Tir-ep, 84 kDa; Tir-ep:GFP, 110 kDa. EspD and EspD:GFP, which were not detected, were predicted at 49 and 76 kDa, respectively (not shown).

As expected, neither EspD nor EspD:GFP were detectable by Western blot (data not shown). The blot did reveal signals for Tir-ep:GFP that were much stronger that the barely detectable signals for Tir-ep, indicating that the GFP fusion improves accumulation of Tir-ep (**Figure 2**). The signal for Tir-ep:GFP presented as a major band between 100 and 150 kDa and one high MW band at around 250 kDa. The expected size of the fusion protein is 110 kDa, based on its amino acid composition and considering cleavage of the transit peptide. The higher band may be a product of protein aggregation.

In contrast to Tir-ep:GFP, the signals for NleA:GFP, EspA:GFP, and Tir:GFP were each weaker than those of the respective unfused protein, suggesting that the GFP fusion reduces accumulation of these recombinant proteins (**Figure 2**). While the dissimilar effects of the GFP fusion are not readily explained, they are not surprising given the existing literature in a variety of systems. For example, a study of multiple recombinant mammalian proteins expressed in *E. coli* with C-terminal GFP fusions found that ten had expression levels that were greater, and seven had expression levels that were lower, than the corresponding unfused protein (Dyson et al., 2004).

For some of our recombinant proteins, there are clear mismatches between the size estimates based on the blot and the predicted sizes based on amino acid composition. For example, EspA runs below 50 kDa while its predicted size is 51 kDa. Such gel shifting is common and can be explained by protein tertiary structure or detergent binding in the polyacrylamide gel. Migration through the gel is influenced by aggregation of SDS molecules at hydrophobic protein sites, and can therefore be affected by different proportions of these sites in proteins of interest compared to polypeptides used in the ladder (Rath et al., 2009).

### GFP Fusion Proteins Localize to Chloroplasts

**FIGURE 3:**
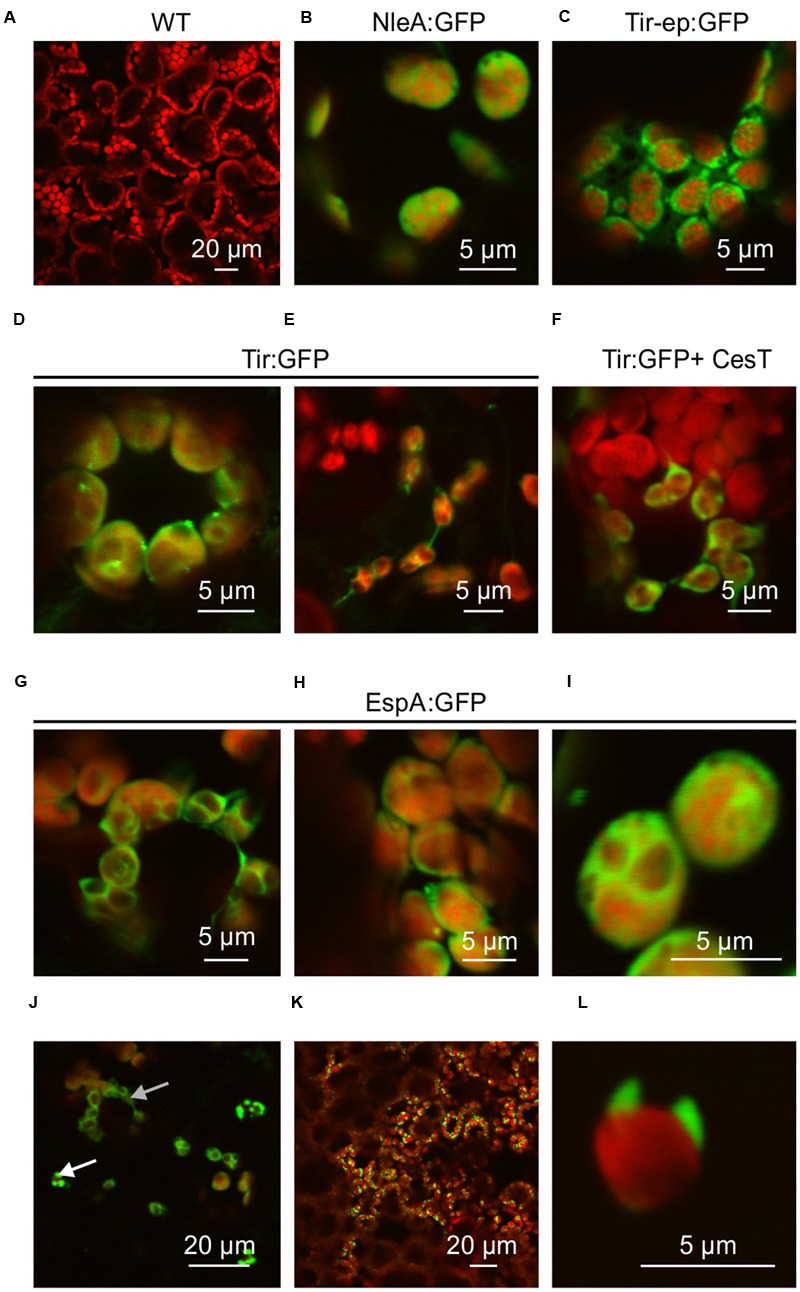
**GFP fusion proteins localize to chloroplasts.** Confocal fluorescence microscopy showing GFP fusion proteins (green) and plant chlorophyll (red). Recombinant proteins were targeted to chloroplasts using the transit peptide from the small subunit of tobacco RuBisCO, and expressed transiently in agro-infiltrated *N. benthamiana* along with the p19 suppressor of gene silencing. **(A)** Wildtype untransformed; **(B)** NleA:GFP; **(C)** Tir-ep:GFP; **(D,E)** Tir:GFP; **(F)** Tir:GFP co-expressed with the T3SS chaperone CesT; **(G–L)** EspA:GFP. Arrows in **(J)** show EspA in the stroma or envelope (top arrow) and in chloroplast-associated protrusions (bottom arrow). Detector sensitivity (HV) was adjusted to best visualize each GFP fusion protein, but was no higher than 500 for GFP acquisition, and images were captured at speeds of 20 or 40 μs/pixel. For the untransformed control, HV was set at 500 and speed at 40 μs/pixel. Scale bars are indicated.

To determine whether the GFP fusion proteins were successfully targeted to chloroplasts, plant samples were viewed using confocal fluorescence microscopy. GFP signals appeared to be associated with the chloroplast stroma, as expected for the chloroplast transit peptide of small subunit RuBisCO which was used in this study, or possibly with the plastid envelope (**Figure 3**). NleA:GFP and Tir-ep:GFP were associated with chloroplast stroma (**Figures 3A,B**). Tir:GFP was sometimes associated with stromules (**Figure 3E**), while EspA:GFP was observed both in the stroma or envelope (**Figures 3G–I**) and frequently in chloroplast-associated protrusions (**Figures 3J–L**).

Both stromules and shorter protrusions occur naturally (Moser et al., 2015) and may be related to multiple biological processes, including photorespiration (Buchner et al., 2015). Chloroplast protrusions also form in response to overexpression of outer- and inner-membrane envelope proteins, in a concentration-dependent manner; possibly reflecting a mechanism to compensate for high amounts of protein while keeping the protein-to-lipid ratio constant over most of the membrane (Machettira et al., 2011). It is therefore possible that EspA:GFP is incompletely imported into the chloroplast, and protrusions result from its association with the membrane-bound import machinery. Alternatively, the EspA:GFP protrusions may result from the propensity of EspA to self-oligomerize; or, given that EspA is implicated in attachment to mammalian intestinal cells (Cleary et al., 2004) and leaf epidermis (Shaw et al., 2008), it may also bind external structures on the chloroplast.

### Co-expression with CesT Chaperone Increases Accumulation of Recombinant Chloroplast-Targeted Tir

**FIGURE 4:**
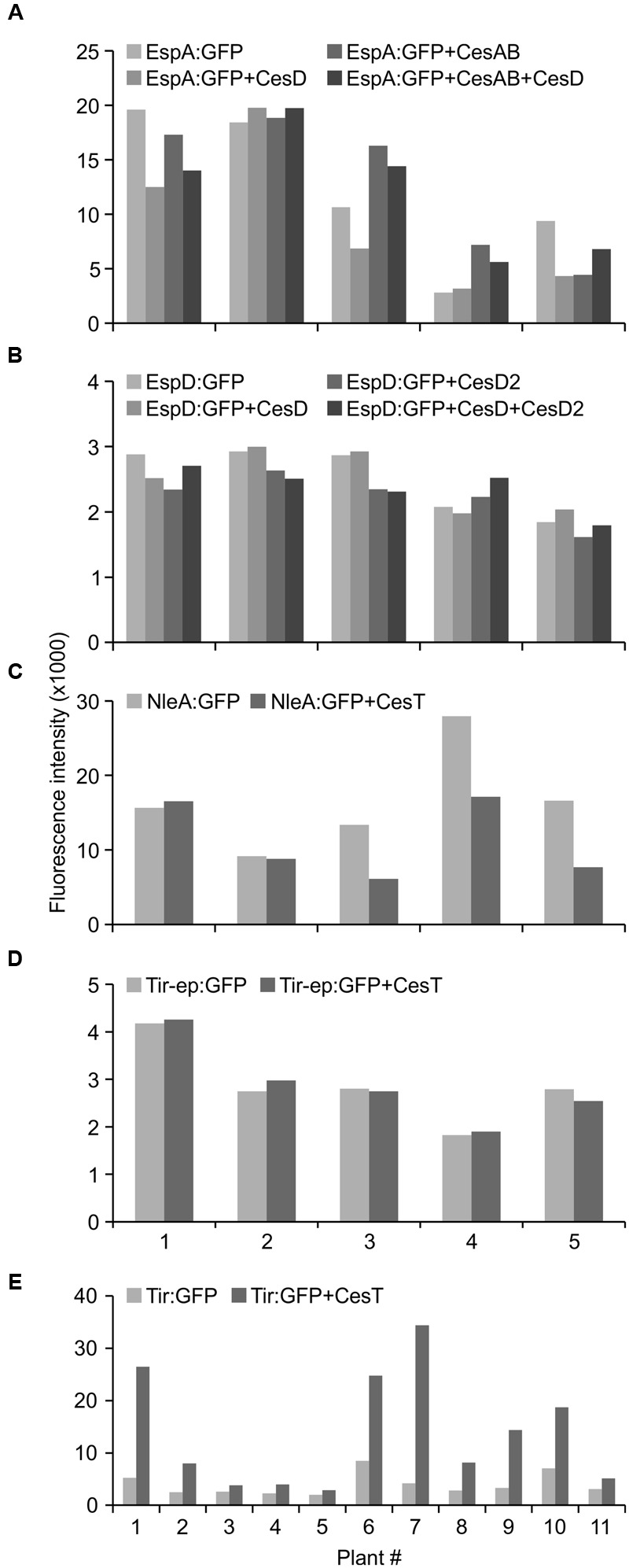
**Co-expression with CesT chaperone increases accumulation of recombinant chloroplast-targeted Tir:GFP.** Relative GFP fluorescence in intact leaf disks expressing proteins of interest (EspA:GFP, EspD:GFP, NleA:GFP, Tir-ep:GFP, Tir:GFP) with or without T3SS chaperones (CesD, CesAB, CesD2, CesT) was measured by fluorescence microtiter plate spectroscopy. Recombinant proteins and chaperones were targeted to the plant chloroplasts using the transit peptide from the small subunit of tobacco RuBisCO, and all infiltrated samples included the p19 suppressor of gene silencing. Infiltrated samples with and without chaperones were included on the same leaf and paired for statistical analysis. *P*-values from paired *t*-tests: **(A)** EspA:GFP vs. EspA:GFP+CesD, 0.1518; EspA:GFP vs. EspA:GFP+CesAB, 0.7618; EspA:GFP vs. EspA:GFP+CesD+CesAB, 0.9788; **(B)** EspD:GFP vs. EspD:GFP+CesD, 0.7955; EspD:GFP vs. EspD:GFP+CesD2, 0.0853; EspD:GFP vs. EspD:GFP+CesD+CesD2, 0.4343; **(C)** NleA:GFP vs. NleA:GFP+CesT, 0.0866; **(D)** Tir-ep:GFP vs. Tir-ep:GFP+CesT, 0.8414; **(E)** Tir:GFP vs. Tir:GFP+CesT, 0.0070. Leaf disks were harvested at 4 dpi. The X-axis indicates different plants as biological replicates.

With the goal of increasing accumulation levels of the recombinant proteins in *Nicotiana* chloroplasts, proteins of interest were co-expressed with select T3SS chaperone proteins (CesAB, CesD, CesD2, or CesT), based on previous findings that indicate positive roles for these chaperones in *E. coli* (**Table 1**). The following co-expressions did not significantly increase accumulation levels of the proteins of interest (*p* > 0.05): EspA:GFP with CesD, CesAB, or both; EspD:GFP with CesD, CesD2, or both; NleA:GFP with CesT; and Tir-ep:GFP with CesT (**Figures 4A–D**), the latter being consistent with the exclusion of the CesT binding domain from the Tir-ep protein.

**FIGURE 5:**
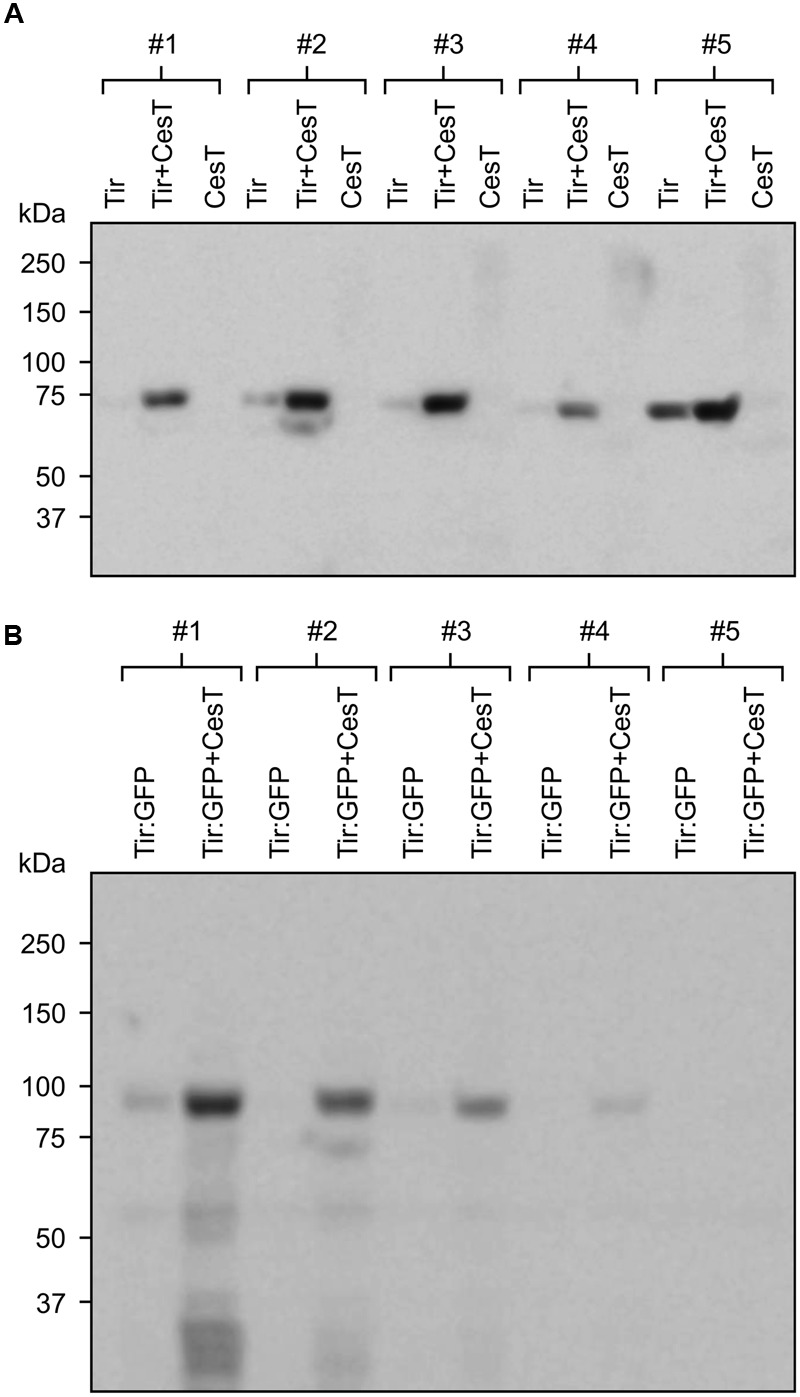
**Co-expression with CesT increases accumulation of recombinant chloroplast-targeted Tir and Tir:GFP.** Relative accumulation of Tir **(A)** and Tir:GFP **(B)** expressed with or without CesT, as determined by Western blot. Proteins were targeted to the plant chloroplasts using the transit peptide from the small subunit of tobacco RuBisCO, and expressed transiently in agro-infiltrated *N. benthamiana* along with the p19 suppressor of gene silencing. For each replicate plant (#1–5), a single leaf was infiltrated with and without CesT on opposite sides of the mid-vein. Infiltrated plant tissue was harvested at 4 dpi. Blots were probed with anti-c-myc antibody. Predicted protein sizes, based on amino acid composition, are: Tir, 68 kDa; Tir:GFP, 95 kDa. The predicted size of CesT (not shown) is 28 kDa.

By contrast, when Tir:GFP and CesT were co-expressed via agroinfiltration of one side of a leaf, and Tir:GFP was expressed by itself on the opposite side of the same leaf, the co-expression always produced a higher amount of Tir:GFP (**Figures 4E**). This difference was very significant as determined by a paired *t*-test (*P* = 0.0070), which is used when observations in one data set are paired with observations in the other data set (here, each replicate sample of Tir:GFP with CesT is paired with a sample of Tir:GFP without CesT that was infiltrated on the same leaf). The same pattern was observed from Western blots of either Tir or Tir:GFP transiently expressed with and without CesT (**Figure 5**). When co-expressed with CesT, Tir:GFP remained associated with the chloroplast (**Figure 3F**).

To study the effect of CesT on accumulation of Tir:GFP over time, leaf tissue from infiltrated plants was harvested at three time points – 4, 6, and 8 dpi – and relative GFP fluorescence was measured on the day of harvest, as well as two days and four days after harvest. While the fluorescence from Tir:GFP expressed without CesT dropped to levels consistent with uninfiltrated WT plants by 6 dpi or 2 days after harvest of the 4 dpi sample, fluorescence from Tir:GFP co-expressed with CesT dropped more slowly (**Table 2**). When co-expressed with CesT, fluorescence from Tir:GFP did not drop to levels of uninfiltrated WT plants over the study period. These results show that CesT can help maintain higher levels of Tir:GFP over time both *in vivo* and *ex vivo*, indicating that the favorable effect of CesT on accumulation of Tir is not specific to a single time point or to fresh material.

**Table 2:** Relative GFP fluorescence in intact leaf disks expressing Tir:GFP with or without CesT over time ± SD, *n* = 6.

		Tir:GFP		
Harvest Time		4 dpi	6 dpi	8 dpi
Post-harvest age	0 days	2806 ± 2166	413 ± 80	460 ± 148
	2 days	311 ± 187	366 ± 157	798 ± 471
	4 days	512 ± 120	955 ± 468	1026 ± 407
		
		**Tir:GFP+CesT**		
		
Post-harvest age	0 days	15545 ± 9916	10357 ± 5055	7390 ± 5189
	2 days	11107 ± 8432	6042 ± 2914	3551 ± 1985
	4 days	6097 ± 6146	3884 ± 2031	2754 ± 1729

*Mean value for uninfiltrated control (2047) has been subtracted from each.*

### Co-expression with CesT Increases Accumulation of Transplastomic Tir

**FIGURE 6:**
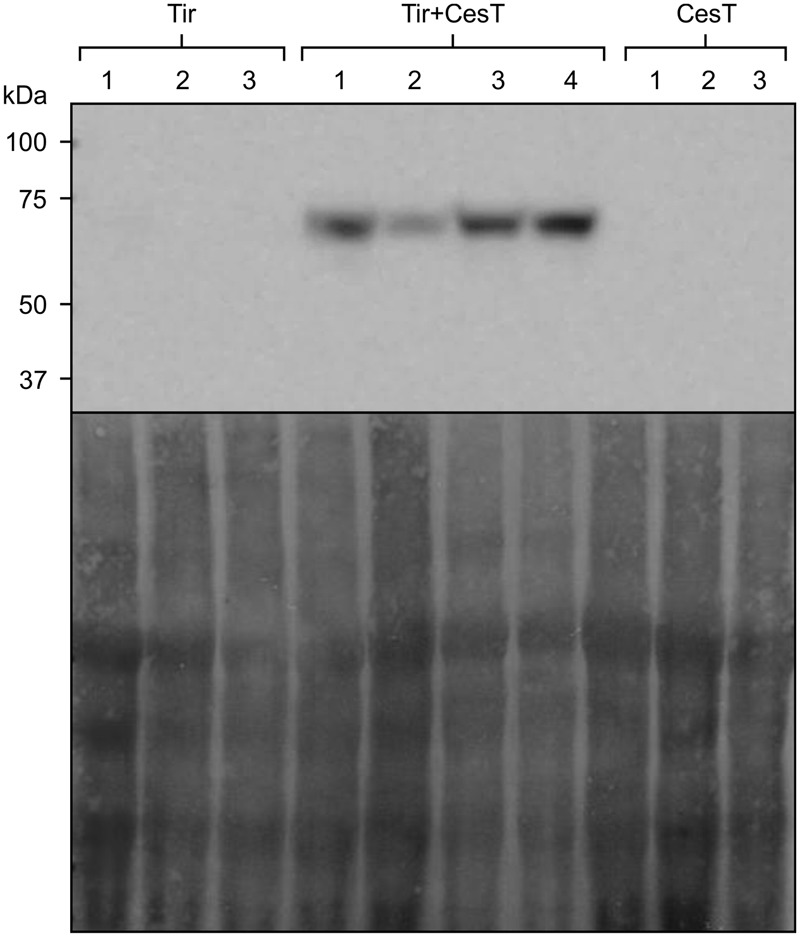
**CesT allows stable accumulation of Tir.** Relative accumulation of transplastomically expressed Tir with or without chloroplast-targeted CesT, as determined by Western blot (upper panel) with loading control shown by staining the membrane (lower panel). The *N. tabacum* chloroplast genome was stably transformed to express Tir, and the nuclear genome of transplastomic plants was stably transformed to express CesT targeted to the plant chloroplasts using the transit peptide from the small subunit of tobacco RuBisCO. Samples of plants transplastomic for *Tir* but without *CesT*, and plants transgenic for *CesT* without transplastomic *Tir*, are indicated. Protein loaded in each well was extracted from an equivalent amount of fresh weight material (4 mg). The predicted size of transplastomic Tir, based on amino acid composition, is 61 kDa. The predicted size of CesT (not shown) is 28 kDa. Numbers above the lanes indicate independent transplastomic plants in the case of Tir, or independent transgenic lines in the case of Tir+CesT and CesT. The Tir+CesT lines were derived from transformation of the same Tir line that is shown in the figure.

To determine whether CesT would also improve accumulation of Tir that is expressed from the chloroplast genome, transplastomic *N. tabacum* plants carrying the *Tir* gene were stably transformed with the construct for chloroplast-targeted CesT. Chloroplast-targeted, nuclear-encoded CesT was used as a model to test effects on transplastomic Tir, with the intent that both proteins could be expressed transplastomically in the future. These doubly transformed plants accumulated Tir to levels detectable by Western blot, while plants without *CesT* did not produce detectable levels of Tir (**Figure 6**).

**FIGURE 7:**
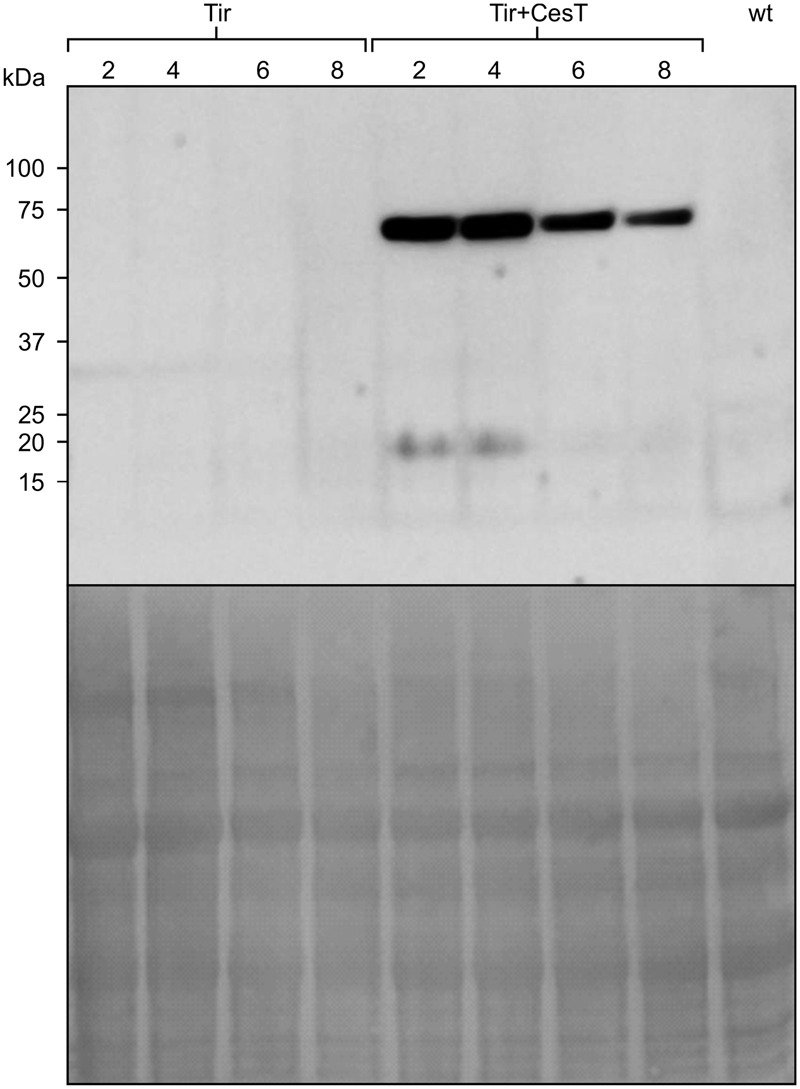
**Tir accumulates in both young and older leaves when co-expressed with CesT.** Relative accumulation of transplastomically expressed Tir with or without chloroplast-targeted CesT. Leaf tissue was sampled from leaves 2, 4, 6, and 8 of mature plants, with 2 being a young leaf and 8 an older leaf. Relative accumulation in each leaf was determined by Western blot (upper panel) with loading control shown by staining the membrane (lower panel). Protein loaded in each well was extracted from an equivalent amount of fresh weight material (11.6 mg). Wt refers to leaf 4 of an untransformed *N. tabacum* plant. The predicted size of transplastomic Tir, based on amino acid composition, is 61 kDa. The predicted size of CesT is 28 kDa, and a band running at about 20 kDa is apparent on the blot in leaves 2 and 4.

Since recombinant protein stability and leaf age are linked, samples of leaves of different ages (**Figure 7**) were taken from the transplastomic *N. tabacum* plants carrying the *Tir* gene with and without chloroplast-targeted CesT. Without CesT, transplastomic Tir was not detected by Western blot. By contrast, in the presence of CesT, the accumulation of Tir was noticeable across all samples, even though older leaves contained less Tir protein, and undetectable CesT. The finding that CesT improves accumulation of transplastomic Tir even in older leaves of *N. tabacum* is consistent with the finding that CesT maintains higher levels of Tir:GFP over time in transiently transformed *N. benthamiana*. It is possible that the lower levels of Tir in older leaves is due to a lower level of CesT in those leaves.

## Discussion

This study found that co-expression of CesT can improve accumulation of Tir in plant chloroplasts, when Tir is either targeted to the chloroplast of *N. benthamiana* or expressed transplastomically in *N. tabacum*.

The finding that accumulation in plant chloroplasts of recombinant Tir can be increased by co-expressing CesT is in agreement with previous studies in EPEC, where intracellular levels of both Tir and NleA are compromised in CesT knockout mutants (Thomas et al., 2005; Ramu et al., 2013). Based on our results that plant-made recombinant NleA accumulation remained unaltered by co-expression with CesT, and that NleA accumulated to relatively high levels on its own, CesT may affect Tir and NleA through different mechanisms. In support of this interpretation, a previous report by Ramu et al. (2013) tested the ability of plasmid-encoded, mutated CesT to rescue phenotypes in their CesT knockout EPEC; and found that some of the CesT mutants restored normal intracellular levels of Tir but not of NleA. It is therefore possible that a role of CesT is to prevent interactions between NleA and certain destabilizing factors present in EPEC, but that such factors are not present in plant chloroplasts. Alternatively, NleA but not Tir may be stabilized by factors present in plant chloroplasts but absent in *E. coli*; or by the additional amino acids added to our constructs (similar to the stabilization of Tir-ep by the C-terminal GFP fusion).

Given that interaction with CesT is known to stabilize Tir in its native pathogenic *E. coli*, this seems to be the most likely mechanism by which CesT improves accumulation of Tir in plant chloroplasts. However, another possibility is that CesT competes with Tir as a proteolysis substrate in chloroplasts. While we think this possibility is slim, it would nonetheless be useful to verify by co-expressing Tir with CesT loss-of-function mutants.

Unlike Tir, the accumulation levels of EspA and EspD were not affected by the chaperones tested in this study. In pathogenic *E. coli*, these proteins are known to be affected by multiple chaperones, not all of which were included here (for example, CesA2 and EscL stabilize EspA; Su et al., 2008; Ku et al., 2009); and some may yet to be discovered. While recombinant EspA and EspA:GFP did express in plants, EspD and EspD:GFP were not detected. Recombinant EspD in *E. coli* has previously been associated with low expression levels and formation of inclusion bodies (Daniell et al., 2001; Dasanayake et al., 2011); characteristics that may not have been rescued by the chaperones. C-terminal GFP fusions are not fluorescent when expressed in inclusion bodies (Drew et al., 2001).

The low accumulation of Tir-ep without the GFP fusion may be due to the presence of multiple transmembrane domains. Tir-ep was engineered by combining the intimin-binding domains of Tir from four EHEC serotypes with the intent of developing a multi-valent subunit vaccine against these serotypes. Prediction of the transmembrane regions using the online TMHMM tool v 2.0 (Krogh et al., 2001) indicated two transmembrane regions within each intimin binding domain for a total of eight for the entire Tir-ep protein (See underlined sequence in Supplementary Data for the domains). It is possible that these regions may contribute to misfolding of the protein, which may prevent its accumulation. On the other hand, its fusion to GFP results in increased accumulation possibly due to the effect of GFP as a soluble partner.

Our findings demonstrate that a T3SS chaperone (CesT) and its substrate (Tir) interact in a heterologous environment to increase accumulation of the substrate protein. This increase in accumulation is evident over time, both *in vivo* and *ex vivo*, whether Tir is expressed from the chloroplast genome or whether it is transported to the plant chloroplast with or without a fusion to GFP. This finding may contribute to the development of stable, transplastomic vaccine candidates against EHEC. Yet while we highlight the potential of chaperones to increase accumulation of recombinant proteins in heterologous systems which may not have similar native chaperones, we also demonstrate that this is not a universal solution. The finding that some chaperone–substrate combinations do not increase accumulation of the T3SS protein in plant chloroplasts has implications for understanding the complexity of T3SS chaperone–substrate interactions in their native system.

## Author Contributions

JM designed and completed most of the experiments and wrote the manuscript. SM designed and cloned some of the constructs. TG contributed to cloning, Western blots, and transformations. AC-F performed Western blots of incremental leaf samples. RM supervised the study and edited the manuscript. All authors read and approved the final manuscript.

## Conflict of Interest Statement

The authors declare that the research was conducted in the absence of any commercial or financial relationships that could be construed as a potential conflict of interest.

## Abbreviations

dpi: days post infiltration
EDTA: ethylenediaminetetraacetic acid
EHEC: enterohaemorrhagic *E. coli*
EPEC: enteropathogenic *E. coli*
EspA: *E. coli* secreted protein A
EspD: *E. coli* secreted protein D
GFP: green fluorescent protein
LEE: locus of enterocyte effacement
NleA: non-LEE encoded factor A
PBS: phosphate-buffered saline
PMSF: phenylmethylsulfonyl fluoride
PVPP: polyvinylpolypyrrolidone
SDS: sodium dodecyl sulfate
T3SS: type 3 secretion system
Tir: translocated Intimin receptor
Tir-ep: fusion of epitopes from Tir sequences
WT: wild type
