# Corrigendum: Co-expression with the Type 3 Secretion Chaperone CesT from Enterohemorrhagic *E. coli* Increases Accumulation of Recombinant Tir in Plant Chloroplasts

**DOI:** 10.3389/fpls.2017.00796

**Published:** 2017-05-12

**Authors:** Jacqueline MacDonald, Sean Miletic, Typhanie Gaildry, Adam Chin-Fatt, Rima Menassa

**Affiliations:** ^1^London Research and Development Centre, Agriculture and Agri-Food CanadaLondon, ON, Canada; ^2^Department of Biology, University of Western OntarioLondon, ON, Canada; ^3^Department of Biology, Université de BordeauxTalence, France

**Keywords:** chloroplast targeting, EHEC, EPEC, fluorescence microscopy, T3SS, translocated intimin receptor, transplastomic, subunit vaccine

In the original article, there was a mistake in the Supplementary Data (Data Sheet 1) as published. The sequences given for RbcS-TP were incorrect, and KDEL sequences were included that should not have been there. The corrected Data Sheet 1 is uploaded.

The authors apologize for this error and state that this does not change the scientific conclusions of the article in any way. The corrected supplementary material has been updated on the original article page.

## Conflict of interest statement

The authors declare that the research was conducted in the absence of any commercial or financial relationships that could be construed as a potential conflict of interest.

