# Supplementary material for: Co-expression with the Type 3 Secretion Chaperone CesT from Enterohemorrhagic E. coli Increases Accumulation of Recombinant Tir in Plant Chloroplasts
Source: Front Plant Sci. 2017 Mar 6;8:283. doi: 10.3389/fpls.2017.00283 (PMC5337511; doi:10.3389/fpls.2017.00283)
Supplement: Supplementary file 1 [file Data_Sheet_1.docx]

**CODING SEQUENCE FOR TRANSPLASTOMIC CONSTRUCTS**

**Transplastomic construct with Tir**

| 5’ seq | Tir | KasI | c-myc | StrepII |
| --- | --- | --- | --- | --- |

***SEQUENCES (TRANSPLASTOMIC)***

**5’ seq**

| nucleotide: | ATGGCTAGC |
| --- | --- |
| amino acid: | MAS |

**Tir**

| nucleotide: | CCAATCGGTAACTTGGGTCATAACCCAAACGTTAACAACTCAATCCCTCCTGCACCACCTTTGCCATCTCAAACAGATGGTGCTGGAGGTAGAGGACAACTTATCAATTCAACTGGACCACTTGGTTCAAGAGCTTTATTCACACCAGTTAGAAACTCAATGGCAGACTCAGGTGACAATAGGGCTTCTGATGTTCCAGGACTTCCAGTAAATCCTATGAGATTGGCAGCATCTGAAATCACACTTAACGACGGTTTCGAAGTTTTGCATGACCACGGTCCATTGGATACTCTTAACAGACAAATCGGAAGTTCGGTTTTCCGAGTTGAAACGCAAGAGGACGGTAAACATATAGCAGTCGGTCAACGAAACGGTGTAGAAACTTCTGTTGTTCTATCAGACCAAGAGTACGCTAGACTACAATCCATTGACCCTGAGGGTAAAGATAAGTTTGTGTTTACTGGTGGAAGGGGTGGAGCTGGTCATGCTATGGTCACAGTTGCATCTGATATTACAGAAGCAAGACAAAGAATACTAGAGTTGTTGGAGCCTAAGGGTACGGGTGAATCAAAGGGTGCTGGTGAAAGTAAAGGAGTTGGAGAACTTCGAGAATCAAACAGTGGAGCTGAAAATACGACGGAAACACAGACATCAACAAGTACCTCAAGTCTACGTTCCGATCCTAAGCTATGGTTGGCACTAGGAACAGTTGCTACAGGATTAATTGGACTAGCAGCAACCGGTATAGTTCAAGCACTTGCATTGACACCTGAACCTGATTCTCCTACTACAACCGATCCAGATGCAGCTGCTTCTGCAACTGAAACTGCTACTCGTGATCAATTAACAAAGGAAGCCTTTCAAAATCCTGATAATCAGAAAGTGAATATAGATGAGTTGGGGAATGCGATTCCCTCGGGAGTCCTTAAAGATGATGTCGTCGCTAATATTGAGGAGCAGGCCAAAGCGGCTGGAGAAGAAGCTAAACAACAAGCTATTGAGAATAATGCGCAGGCTCAGAAAAAATATGATGAGCAGCAGGCGAAACGACAGGAAGAACTTAAAGTGTCGTCGGGAGCTGGATATGGACTTTCGGGAGCCTTAATTTTAGGAGGCGGAATAGGAGTAGCTGTAACAGCTGCCTTACATCGAAAAAATCAACCTGTGGAACAAACAACCACTACCACTACTACCACAACTACGACGTCGGCTCGAACTGTAGAAAATAAGCCGGCTAATAATACCCCCGCTCAAGGGAATGTAGATACTCCTGGCTCTGAAGATACTATGGAATCGAGGCGTTCTTCTATGGCTTCAACTAGTTCCACTTTCTTTGATACCTCCTCCATTGGGACAGTACAAAATCCCTATGCTGATGTAAAAACCTCCTTACACGATTCCCAAGTACCCACCTCCAATTCCAATACCTCCGTGCAAAATATGGGGAATACCGATTCTGTAGTATATTCTACTATTCAACACCCGCCGCGTGATACTACTGATAATGGCGCGAGGTTATTAGGGAATCCCTCTGCCGGCATTCAAAGTACTTATGCCCGATTAGCCTTAAGTGGGGGGTTACGTCATGATATGGGCGGGTTAACTGGGGGGTCTAATAGTGCCGTAAATACTTCTAATAATCCGCCTGCCCCCGGCAGTCATCGTTTTGTA |
| --- | --- |
| amino acid: | PIGNLGHNPNVNNSIPPAPPLPSQTDGAGGRGQLINSTGPLGSRALFTPVRNSMADSGDNRASDVPGLPVNPMRLAASEITLNDGFEVLHDHGPLDTLNRQIGSSVFRVETQEDGKHIAVGQRNGVETSVVLSDQEYARLQSIDPEGKDKFVFTGGRGGAGHAMVTVASDITEARQRILELLEPKGTGESKGAGESKGVGELRESNSGAENTTETQTSTSTSSLRSDPKLWLALGTVATGLIGLAATGIVQALALTPEPDSPTTTDPDAAASATETATRDQLTKEAFQNPDNQKVNIDELGNAIPSGVLKDDVVANIEEQAKAAGEEAKQQAIENNAQAQKKYDEQQAKRQEELKVSSGAGYGLSGALILGGGIGVAVTAALHRKNQPVEQTTTTTTTTTTTSARTVENKPANNTPAQGNVDTPGSEDTMESRRSSMASTSSTFFDTSSIGTVQNPYADVKTSLHDSQVPTSNSNTSVQNMGNTDSVVYSTIQHPPRDTTDNGARLLGNPSAGIQSTYARLALSGGLRHDMGGLTGGSNSAVNTSNNPPAPGSHRFV |

**KasI**

| nucleotide: | GGCGCC |
| --- | --- |
| amino acid: | GA |

**c-myc**

| nucleotide: | GAACAAAAATTGATTTCGGAAGAAGATCTA |
| --- | --- |
| amino acid: | EQKLISEEDL |

**StrepII**

| nucleotide: | TGGTCGCATCCTCAATTTGAAAAATAA |
| --- | --- |
| amino acid: | WSHPQFEK• |

**CODING SEQUENCE FOR TRANSIENT / NUCLEAR CONSTRUCTS**

**Transient/nuclear constructs with GOI**

| RbcS-TP | Xpress | attB1 | GOI | XbaI | StrepII | attB2 | c-myc |
| --- | --- | --- | --- | --- | --- | --- | --- |

**Transient/nuclear constructs with GOI-GFP fusion**

| RbcS-TP | Xpress | attB1 | GOI | XbaI | GFP | XbaI | StrepII | attB2 | c-myc |
| --- | --- | --- | --- | --- | --- | --- | --- | --- | --- |

**Transient/nuclear constructs with CesT**

| RbcS-TP | Xpress | attB1 | CesT | attB2 | c-myc |
| --- | --- | --- | --- | --- | --- |

***VECTOR SEQUENCES (TRANSIENT / NUCLEAR)***

**RbcS-TP**

| nucleotide: | ATGGCTTCCTCAGTTCTTTCCTCTGCAGCAGTTGCCACCCGCAGCAATGTTGCTCAAGCTAACATGGTTGCACCTTTCACTGGCCTTAAGTCAGCTGCCTCATTCCCTGTTTCAAGGAAGCAAAACCTTGACATCACTTCCATTGCCAGCAACGGCGGAAGAGTGCAATGC |
| --- | --- |
| amino acid: | MASSVLSSAAVATRSNVAQANMVAPFTGLKSAASFPVSRKQNLDITSIASNGGRVQC |

**Xpress**

| nucleotide: | GATCTCTATGATGACGATGACAAA |
| --- | --- |
| amino acid: | DLYDDDDK |

**attB1**

| nucleotide: | GTTATCGTTATCACAAGTTTGTACAAAAAAGCAGGCTTG |
| --- | --- |
| amino acid: | VIVITSLYKKAGL |

**GOI**

*variable* (see below)

**Xba1**

| nucleotide: | TCTAGA |
| --- | --- |
| amino acid: | SR |

**StrepII**

| nucleotide: | TGGTCACATCCTCAATTTGAGAAA |
| --- | --- |
| amino acid: | WSHPQFEK |

**attB2**

| nucleotide: | GACCCAGCTTTCTTGTACAAAGTGGTGATAACT |
| --- | --- |
| amino acid: | DPAFLYKVVIT |

**c-myc**

| nucleotide: | GAACAAAAGTTGATCTCTGAGGAAGACCTC |
| --- | --- |
| amino acid: | EQKLISEEDL• |

***REPORTER SEQUENCES (TRANSIENT / NUCLEAR)***

**GFP**

| nucleotide: | ATGGCTAGCGTGAGCAAGGGCGAGGAGCTGTTCACCGGGGTGGTGCCCATCCTGGTCGAGCTGGACGGCGACGTAAACGGCCACAAGTTCAGCGTGTCCGGCGAGGGCGAGGGCGATGCCACCTACGGCAAGCTGACCCTGAAGTTCATCTGCACCACCGGCAAGCTGCCCGTGCCCTGGCCCACCCTCGTGACCACCCTGACCTACGGCGTGCAGTGCTTCAGCCGCTACCCCGACCACATGAAGCAGCACGACTTCTTCAAGTCCGCCATGCCCGAAGGCTACGTCCAGGAGCGCACCATCTTCTTCAAGGACGACGGCAACTACAAGACCCGCGCCGAGGTGAAGTTCGAGGGCGACACCCTGGTGAACCGCATCGAGCTGAAGGGCATCGACTTCAAGGAGGACGGCAACATCCTGGGGCACAAGCTGGAGTACAACTACAACAGCCACAACGTCTATATCATGGCCGACAAGCAGAAGAACGGCATCAAGGTGAACTTCAAGATCCGCCACAACATCGAGGACGGCAGCGTGCAGCTCGCCGACCACTACCAGCAGAACACCCCCATCGGCGACGGCCCCGTGCTGCTGCCCGACAACCACTACCTGAGCACCCAGTCCGCCCTGAGCAAAGACCCCAACGAGAAGCGCGATCACATGGTCCTGCTGGAGTTCGTGACCGCCGCCGGGATCACTCTCGGCATGGACGAGCTGTACAAG |
| --- | --- |
| amino acid: | MASVSKGEELFTGVVPILVELDGDVNGHKFSVSGEGEGDATYGKLTLKFICTTGKLPVPWPTLVTTLTYGVQCFSRYPDHMKQHDFFKSAMPEGYVQERTIFFKDDGNYKTRAEVKFEGDTLVNRIELKGIDFKEDGNILGHKLEYNYNSHNVYIMADKQKNGIKVNFKIRHNIEDGSVQLADHYQQNTPIGDGPVLLPDNHYLSTQSALSKDPNEKRDHMVLLEFVTAAGITLGMDELYK |

***GOI SEQUENCES (TRANSIENT / NUCLEAR)***

***T3SS PROTEINS AND EFFECTORS***

**NleA**

| nucleotide: | ATGAACATCCAGCCAACGATCCAATCCGGAATCACAAGTCAAAACAACCAGCACCATCAGACAGAGCAAATCCCTTCAACACAAATCCCGCAAAGTGAGCTACCACTAGGATGTCAAGCAGGTTTTGTAGTCAACATCCCTGATGATATCCAACAGCACGCTCCAGAATGCGGAGAAACAACAGCATTGCTTTCATTGATCAAGGACAAGGGACTACTAAGTGGTCTGGATGAATACATTGCTCCACACCTGGAAGAAGGATCAATTGGAAAGAAGACGCTGGATATGTTCGGATTATTCAACGTCACCCAGATGGCTCTGGAAATTCCAAGTAGCGTCTCAGGTATTAGCGGAAAGTACGGAGTTCAGCTTAACATTGTCAAGCCAGACATCCACCCAACTTCAGGTAACTATTTCCTCCAGATATTCCCACTCCACGATGAAATTGGTTTCAACTTCAAGGACCTCCCAGGACCTTTGAAGAACGCACTCTCTAATAGCAATATTAGCACCACCGCAGTATCCACGATTGCATCCACAGGAACATCTGCTACTACATCTACAGTGACAACGGAACCAAAGGACCCAATACCATGGTTCGGTTTGACAGCACAAGTTGTTAGAAATCACGGAGTGGAATTGCCTATAGTGAAAACCGAGAATGGCTGGAAACTTGTGGGAGAAACCCCTCTTACTCCTGATGGTCCTAAAGCAAATTACACCGAAGAATGGGTTATAAGGCCTGGTGAAGCAGACTTCAAATACGGTGCTTCTCCTCTCCAAGCAACTCTTGGTCTTGAATTTGGTGCACATTTTAAATGGGACTTGGACAATCCGAATACCAAATATGCCGTGCTTACTAATGCTGCTGCCAATGCTTTGGGGGCTCTTGGTGGTTTTGCTGTGAGTCGATTTGCTTCTACTGATCCTATGCTTTCCCCTCATATAGGGGCTATGGTAGGTCAAGCTGCAGGCCATGCAATTCAATATAATACTCCTGGCTTAAAACCGGATACTATATTATGGTGGGCCGGGGCTACTTTAGGCGCTGCTGATTTGAACAAAGCTGAGTTTGAGGTTGCCCGTTTTACTGATTATCCTCGTATTTGGTGGCATGCTAGAGAGGGGGCCATTTTTCCCAATAAGGCCGATATAGAGCATGCAACTGGCGCCGATATTAGAGCCATGGAGGAGGGGATTCCCGTTGGGCAGAGGCATCCCAATCCCGAGGATGTTGTAATAGATATTGAGTCTAATGGCTTACCGCATCATAATCCGTCTAATCATGTTGATATTTTTGATATTATTCAGGAGACTCGGGTT |
| --- | --- |
| amino acid: | MNIQPTIQSGITSQNNQHHQTEQIPSTQIPQSELPLGCQAGFVVNIPDDIQQHAPECGETTALLSLIKDKGLLSGLDEYIAPHLEEGSIGKKTLDMFGLFNVTQMALEIPSSVSGISGKYGVQLNIVKPDIHPTSGNYFLQIFPLHDEIGFNFKDLPGPLKNALSNSNISTTAVSTIASTGTSATTSTVTTEPKDPIPWFGLTAQVVRNHGVELPIVKTENGWKLVGETPLTPDGPKANYTEEWVIRPGEADFKYGASPLQATLGLEFGAHFKWDLDNPNTKYAVLTNAAANALGALGGFAVSRFASTDPMLSPHIGAMVGQAAGHAIQYNTPGLKPDTILWWAGATLGAADLNKAEFEVARFTDYPRIWWHAREGAIFPNKADIEHATGADIRAMEEGIPVGQRHPNPEDVVIDIESNGLPHHNPSNHVDIFDIIQETRV |

**Tir**

| nucleotide: | ATGCCAATCGGAAACCTAGGACACAACCCAAACGTAAATAACTCCATCCCCCCAGCTCCTCCTTTACCTAGTCAAACGGACGGAGCTGGAGGTAGAGGTCAATTGATAAACTCTACCGGACCATTAGGAAGTAGAGCATTGTTTACGCCAGTCAGAAACAGTATGGCTGACAGTGGAGACAACCGTGCCAGTGATGTTCCAGGATTACCAGTTAACCCAATGAGACTGGCTGCAAGTGAAATCACTCTAAACGACGGATTCGAGGTACTGCATGATCACGGACCACTTGATACTCTAAATCGTCAGATCGGTTCCTCCGTTTTCAGAGTCGAGACTCAGGAAGACGGTAAACACATCGCTGTAGGACAAAGAAACGGAGTAGAGACGAGTGTCGTTCTATCCGACCAAGAATACGCAAGGCTTCAGAGTATAGACCCAGAGGGAAAGGATAAGTTTGTCTTTACGGGGGGAAGAGGTGGAGCTGGTCATGCAATGGTTACTGTTGCATCTGATATTACGGAGGCTAGACAGCGTATACTGGAATTACTGGAGCCTAAGGGTACGGGAGAATCTAAGGGAGCAGGAGAATCAAAGGGTGTTGGTGAACTTCGAGAAAGTAACTCCGGTGCTGAAAACACAACCGAAACACAGACATCCACCAGTACCAGCTCTTTGCGATCAGATCCTAAACTGTGGTTAGCACTCGGTACAGTAGCTACAGGTTTGATAGGTCTCGCAGCAACTGGTATAGTTCAGGCACTCGCATTGACACCTGAACCAGATTCACCTACAACAACCGATCCAGATGCAGCCGCCTCAGCTACTGAAACTGCAACTAGGGACCAATTGACAAAGGAAGCTTTCCAGAACCCTGATAACCAGAAGGTGAATATTGATGAGCTTGGCAATGCAATTCCTAGCGGTGTGCTCAAGGATGATGTCGTGGCTAATATTGAGGAGCAGGCAAAAGCAGCTGGTGAAGAAGCTAAACAGCAGGCTATTGAGAATAATGCACAGGCACAAAAGAAGTACGATGAGCAACAAGCCAAAAGGCAAGAGGAGCTTAAAGTTAGCTCCGGGGCTGGTTACGGTCTCTCAGGTGCTCTTATTTTGGGAGGTGGTATTGGGGTTGCTGTTACCGCTGCCCTTCATCGTAAAAATCAACCCGTTGAACAGACTACCACAACCACTACAACCACAACTACCACTTCCGCCAGGACAGTCGAAAATAAGCCTGCTAATAATACCCCTGCCCAAGGCAATGTTGACACTCCGGGGTCAGAGGATACTATGGAGTCTAGGCGATCTTCAATGGCCTCAACATCTTCTACATTTTTCGATACTAGCTCAATTGGCACCGTTCAAAATCCGTATGCTGATGTGAAAACAAGCCTCCACGATAGCCAGGTGCCTACAAGCAATAGCAATACATCTGTGCAGAATATGGGGAATACTGATTCTGTGGTGTATTCTACTATTCAACATCCGCCGCGGGATACTACTGATAATGGGGCTAGGTTGTTGGGGAATCCCTCTGCCGGGATTCAATCAACATATGCCAGGCTTGCTCTTTCTGGCGGCCTTCGGCATGATATGGGCGGCTTGACTGGGGGCTCTAATTCAGCTGTGAATACTTCTAATAATCCGCCCGCTCCTGGGTCACATCGGTTTGTT |
| --- | --- |
| amino acid: | MPIGNLGHNPNVNNSIPPAPPLPSQTDGAGGRGQLINSTGPLGSRALFTPVRNSMADSGDNRASDVPGLPVNPMRLAASEITLNDGFEVLHDHGPLDTLNRQIGSSVFRVETQEDGKHIAVGQRNGVETSVVLSDQEYARLQSIDPEGKDKFVFTGGRGGAGHAMVTVASDITEARQRILELLEPKGTGESKGAGESKGVGELRESNSGAENTTETQTSTSTSSLRSDPKLWLALGTVATGLIGLAATGIVQALALTPEPDSPTTTDPDAAASATETATRDQLTKEAFQNPDNQKVNIDELGNAIPSGVLKDDVVANIEEQAKAAGEEAKQQAIENNAQAQKKYDEQQAKRQEELKVSSGAGYGLSGALILGGGIGVAVTAALHRKNQPVEQTTTTTTTTTTTSARTVENKPANNTPAQGNVDTPGSEDTMESRRSSMASTSSTFFDTSSIGTVQNPYADVKTSLHDSQVPTSNSNTSVQNMGNTDSVVYSTIQHPPRDTTDNGARLLGNPSAGIQSTYARLALSGGLRHDMGGLTGGSNSAVNTSNNPPAPGSHRFV |

**Tir-ep**

| nucleotide: | ATGCGGAGTGATCCTAAGCTGTGGTTAGCTCTGGGTACTGTAGCAACAGGACTAATCGGTCTGGCAGCAACAGGAATTGTACAGGCTCTTGCACTAACTCCTGAACCAGATAGTCCAACTACAACAGATCCAGACGCAGCTGCATCTGCAACAGAAACAGCAACAAGAGATCAGCTAACTAAGGAAGCATTCCAGAACCCAGATAACCAGAAGGTCAACATCGACGAGCTGGGTAACGCAATCCCTAGTGGAGTCTTGAAGGATGATGTGGTCGCAAACATCGAAGAGCAAGCAAAGGCTGCAGGAGAAGAAGCTAAGCAACAGGCTATCGAAAACAACGCTCAGGCTCAGAAGAAGTACGACGAACAGCAGGCAAAGAGACAGGAAGAACTGAAGGTCAGTTCCGGTGCTGGTTACGGATTGTCTGGTGCACTAATCCTAGGAGGAGGCATAGGAGTTGCAGTAACAGCAGCATTACACAGAAAGAACCAGCCAGTCGAACAGACAACCACAACCACGACGACAACTACGACAACAAGTGCTGGATCCGGAGGAAGTGGAAGATCAGATCCTAAGTTCTGGGTGTCAATCGGAGCTATCGCAGCAGGATTGGCTGGACTTGCTGCAACAGGAATAACACAAGCTTTAGCACTGACTCCAGAACCAGATGATCCAACGACTACAGATCCAGAACAGGCTGCATCAGCTGCAGAATCTGCTACAAGAGATCAACTCACACAGGAAGCTTTTAAGAATCCTGAGAATCAGAAGGTGAGCATCGACGAGATCGGGAATAGCATCCCGTCCGGAGAACTCAAGGACGATGTGGTGGCTAAGATAGAGGAGCAGGCAAAGGAAGCTGGTGAAGCTGCTAGACAACAAGCTGTTGAATCCAATGCACAGGCACAGCAAAGATACGACACACAGTACGCAAGGCGTCAAGAAGAACTCGAACTCTCCTCAGGAATAGGTTACTCCCTCAGCTCAGCATTGATAGTGGGAGGCGGCATTGGTGCAGGAGTTACAACGGCTCTCCATAGGCGAAATCAACCAGCTGAACAAACAACGACAACTACCACCCATACCGGATCTGGTGGTAGTGGTAGGGCAGATCCTAAACTTTGGCTTAGCCTTGGTACTATTGCTGCTGGTCTTATTGGTATGGCAGCTACCGGTATTGCTCAGGCAGTTGCTCTTACCCCAGAACCTGATGATCCTATTACCACCGATCCTGATGCAGCAGCTAATACCGCAGAAGCAGCAGCTAAAGATCAGCTTACCAAGGAAGCTTTTCAGAACCCTGACAATCAGAAGGTTAATATTGACGAGAACGGAAATGCCATACCGAGCGGGGAGTTGAAAGACGACGTGGTTGCACAGATTGCTGAGCAGGCTAAAGCTGCTGGTGAACAGGCTCGACAAGAAGCTATTGAGAGCAATTCTCAGGCTCAGCAAAAATATGACGAGCAACACGCCAAAAGGGAGCAAGAGATGTCTCTCTCATCAGGGGTAGGTTATGGAATATCAGGGGCTCTTATACTTGGGGGAGGTATAGGTGCTGGTGTTACCGCTGCTTTACATCGTAAGAATCAACCCGCTGAACAAACCATTACCACTAGGACTGGGTCAGGTGGTTCTGGTCGTGCTGATCCGAAACTTTGGTTGTCTTTGGGTACTATTGCCGCTGGTTTGATTGGGATGGCTGCTACTGGCATTGCTCAAGCTGTTGCTCTTACTCCTGAGCCTGATGATCCGACTACGACGGATCCTGATACTGCTGCCTCTACTGCCGAGGCCGCCACTAAAGATCGATTGACTCAAGAGGCCTTCCAAGATCCCGATAAACAAAAAGTAAATATTGATGAGAATGGCAATGCCATTCCCTCTGGCGAGTTGATTGACGACGTTGTTGCCCAAATAGCCGAGCAAGCCAAAGCCGCCGGGGAGCAAGCCCGTCAAGAGGCCATTGAGTCTAATTCACAAGCCCAGAAAAAATATGATGAGCAACACGCTAAAAGGGAACAAGAGATGAGTTTGTCTTCTGGCGTTGGCTATGGCATTAGTGGCGCCTTGATTTTAGGCGGGGGCATTGGGGCCGGGGTTACTGCCGCCTTACATCGGAAAAATCAACCCGCCGAGCAAACTATTACTACTCGGACT |
| --- | --- |
| amino acid: | Tir-ep consists of epitopes in the intimin-binding domain of Tir from various EHEC serotypes (O157:H7, O111:H8, O121:H19, O26:H11, and O45:H2).  red = O157:H7  green = O111:H8 / O121:H19 (same sequence)  orange = O26:H11  purple = O45:H2  GSGGSG = flexible linker  Underlined: Predicted transmembrane domain regions  MRSDPKLWLALGTVATGLIGLAATGIVQALALTPEPDSPTTTDPDAAASATETATRDQLTKEAFQNPDNQKVNIDELGNAIPSGVLKDDVVANIEEQAKAAGEEAKQQAIENNAQAQKKYDEQQAKRQEELKVSSGAGYGLSGALILGGGIGVAVTAALHRKNQPVEQTTTTTTTTTTTSAGSGGSGRSDPKFWVSIGAIAAGLAGLAATGITQALALTPEPDDPTTTDPEQAASAAESATRDQLTQEAFKNPENQKVSIDEIGNSIPSGELKDDVVAKIEEQAKEAGEAARQQAVESNAQAQQRYDTQYARRQEELELSSGIGYSLSSALIVGGGIGAGVTTALHRRNQPAEQTTTTTTHTGSGGSGRADPKLWLSLGTIAAGLIGMAATGIAQAVALTPEPDDPITTDPDAAANTAEAAAKDQLTKEAFQNPDNQKVNIDENGNAIPSGELKDDVVAQIAEQAKAAGEQARQEAIESNSQAQQKYDEQHAKREQEMSLSSGVGYGISGALILGGGIGAGVTAALHRKNQPAEQTITTRTGSGGSGRADPKLWLSLGTIAAGLIGMAATGIAQAVALTPEPDDPTTTDPDTAASTAEAATKDRLTQEAFQDPDKQKVNIDENGNAIPSGELIDDVVAQIAEQAKAAGEQARQEAIESNSQAQKKYDEQHAKREQEMSLSSGVGYGISGALILGGGIGAGVTAALHRKNQPAEQTITTRT |

**EspA**

| nucleotide: | ATGGACACAAGTAACGCTACAAGTGTCGTAAACGTATCAGCTTCTAGTAGTACATCAACAATCTACGACCTAGGTAACATGTCAAAGGATGAAGTAGTAAAGCTATTCGAGGAGCTAGGAGTTTTCCAAGCTGCTATTCTGATGTTCAGTTACATGTACCAAGCTCAATCCAACCTTAGTATCGCTAAGTTCGCTGATATGAACGAGGCAAGTAAGGCTTCTACAACTGCTCAAAAAATGGCAAACCTTGTTGATGCTAAGATCGCAGATGTTCAATCCAGTACAGATAAGAACGCAAAGGCAAAGCTGCCACAAGATGTTATCGATTACATCAACGATCCTAGAAACGATATCTCCGTTACAGGTATACGAGATCTGTCAGGAGACTTAAGTGCAGGAGATTTGCAAACAGTTAAGGCAGCAATATCAGCAAAGGCAAACAACCTCACAACTGTTGTTAATAATAGCCAGCTCGAGATTCAACAAATGTCCAATACCCTGAATCTCCTCACTTCCGCTAGATCAGATGTTCAAAGCCTCCAATATAGGACTATATCAGCTATTTCCCTTGGAAAGGGATCTGGTGGTTCTGGTATGGATACTTCAACTGCAACTTCAGTTGCTTCAGCAAATGCTTCTACTTCTACCTCTACTGTTTACGACTTAGGTTCTATGTCTAAGGATGAAGTGGTGCAACTTTTTAATAAGGTGGGGGTCTTTCAAGCAGCATTGTTGATGTTTGCATATATGTATCAGGCCCAGTCAGACTTGTCTATTGCTAAATTTGCCGACATGAATGAGGCTAGTAAAGAGAGCACTACCGCTCAGAAAATGGCTAATTTGGTCGATGCTAAAATAGCCGATGTGCAGTCATCCTCCGATAAAAATAAGAAAGCCAAATTGCCGCAGGAAGTGATAGACTATATTAATGACCCCCGGAATGACATTACGGTGAGCGGGATTAGCGATCTTAATGCTGAACTTGGGGCTGGCGATTTGCAGACTGTCAAAGCCGCCATTTCTGCCAAATCTAATAATTTGACCACGGTGGTCAATAATTCTCAGCTTGAAATTCAGCAGATGTCTAATACGTTAAATTTGCTTACCAGCGCCCGTTCTGATATTCAGTCTTTACAGTATAGGACCATTAGCGCCATTAGCTTAGGCAAA |
| --- | --- |
| amino acid: | EspA consists of a fusion of EspA from EHEC serotypes O157:H7 and O26H11.  red = O157:H7  orange = O26:H11  GSGGSG = flexible linker  MDTSNATSVVNVSASSSTSTIYDLGNMSKDEVVKLFEELGVFQAAILMFSYMYQAQSNLSIAKFADMNEASKASTTAQKMANLVDAKIADVQSSTDKNAKAKLPQDVIDYINDPRNDISVTGIRDLSGDLSAGDLQTVKAAISAKANNLTTVVNNSQLEIQQMSNTLNLLTSARSDVQSLQYRTISAISLGKGSGGSGMDTSTATSVASANASTSTSTVYDLGSMSKDEVVQLFNKVGVFQAALLMFAYMYQAQSDLSIAKFADMNEASKESTTAQKMANLVDAKIADVQSSSDKNKKAKLPQEVIDYINDPRNDITVSGISDLNAELGAGDLQTVKAAISAKSNNLTTVVNNSQLEIQQMSNTLNLLTSARSDIQSLQYRTISAISLGK |

**EspD**

| nucleotide: | ATGCTAAACGTCAACAACGACACGCTGTCAGTCACTAGTGGAGTAAACACGGCTTCAGGTACATCTGGTATCACTCAATCCGAAACTGGACTGTCTCTGGATCTACAACTAGTCAAGTCCATGAATAGCTCCGCTGGATGGACAGAATCATCACCTTTGCCAACTCCTCCAGCTGGTCATTCTTTGGTTACTCCAAGTGCTGCTGAAGATGTATTAAGTAAGCTATTCGGTGGAATCTCCGGTGAAGTTACAAGTCGAACAGAAGAAGCTGAACCACAGAGAACTAGTTACCCTTACTTGAGCCAGGTTAACACAGTGGACCCTCAACAAATGATGATGATGGTCACGCTGCTTTCATTAGACACCTCCGCTCAAAAGGTATCCTCACTGAAGAATAGCAACGAGATCTACATGGATGGTCAGACCAAGGCACTTGAGAATAAGACCCAGGAATATAAGAAGCAGCTCGAGGAACAACAGAAGGCAGAAGAAAAGAGCCAGAAGAGCAAAATAGTGGGGCAGGTATTCGGATGGCTCGGAGTTGCTCTTACTGCAGTTGCAGCAGTGTTTAATCCCGCTTTATGGGCAGTAGTAGCTATAGGAGCAACAGCAATGGCATTACAAACTGCTGTTGATGTTATGGGAGAAAATGCACCGCAAGGATTGAAAACTGCAGCACAAGTTTTCGGTGGTATTAGCATGGCTGCATCAATTCTCACAGCAGGTGTTGGCGGCGTTTCAAGTTTATTGTCAAAATTTGGCAATGTCGCCAACAAAATTGGCTCCAGCGTTGTGAAAGTGGTGGAGAAGGCTGCAGAAGCTTTGGTGAAAAATGTGTTTGCCAAAATTAGTACCGTCGCTGAGGGTGTTACAAATGGGATTCGTTCTGCTGGGACTACCGCTCTTAATAATGAGGCTGCTCAGCTTCAGATGTTGTCACAGTTGGCTGCCTTTGCCGTTCAAAATCTCACCAGACAAAGTGAGTCTCTTGGGGAGAGTGCCAAACTTGAGCTTGATAAAGCCGCCTCTGAGCTTCAAAACCAAGCCTCTTATCTTCAATCTGTTTCTCAGTTGATGTCTGATTCTGCCAGGGTTAATTCTCGGATAGTGTCTGGGAGGATT |
| --- | --- |
| amino acid: | MLNVNNDTLSVTSGVNTASGTSGITQSETGLSLDLQLVKSMNSSAGWTESSPLPTPPAGHSLVTPSAAEDVLSKLFGGISGEVTSRTEEAEPQRTSYPYLSQVNTVDPQQMMMMVTLLSLDTSAQKVSSLKNSNEIYMDGQTKALENKTQEYKKQLEEQQKAEEKSQKSKIVGQVFGWLGVALTAVAAVFNPALWAVVAIGATAMALQTAVDVMGENAPQGLKTAAQVFGGISMAASILTAGVGGVSSLLSKFGNVANKIGSSVVKVVEKAAEALVKNVFAKISTVAEGVTNGIRSAGTTALNNEAAQLQMLSQLAAFAVQNLTRQSESLGESAKLELDKAASELQNQASYLQSVSQLMSDSARVNSRIVSGRI |

***T3SS CHAPERONES***

**CesT**

| nucleotide: | ATGAGTAGCAGATCAGAGCTTCTATTGGAAAAGTTCGCAGAAAAGATCGGAATCGGATCAATAAGTTTCAACGAAAACAGGCTGTGTTCTTTCGCAATTGACGAAATCTACTACATTAGCTTATCCGACGCAAACGATGAATACATGATGATATATGGTGTCTGCGGAAAGTTTCCTACTGATAACTCCAATTTTGCACTGGAAATTCTCAATGCTAATCTCTGGTTTGCTGAGAATGGTGGTCCATATCTCTGTTATGAAGCTGGGGCTCAATCTTTGTTGTTAGCCCTTCGATTTCCGCTTGATGATGCTACACCCGAAAAGCTAGAAAATGAAATAGAGGTAGTTGTGAAATCTATGGAGAATCTTTACTTAGTTCTTCATAATCAGGGCATTACCTTGGAGAATGAGCACATGAAAATTGAGGAGATTTCAAGTTCTGATAACAAACATTATTATGCCGGCCGT |
| --- | --- |
| amino acid: | MSSRSELLLEKFAEKIGIGSISFNENRLCSFAIDEIYYISLSDANDEYMMIYGVCGKFPTDNSNFALEILNANLWFAENGGPYLCYEAGAQSLLLALRFPLDDATPEKLENEIEVVVKSMENLYLVLHNQGITLENEHMKIEEISSSDNKHYYAGR |

**CesAB**

| nucleotide: | ATGTCCATCGTATCTCAAACAAGAAACAAGGAGCTTCTGGATAAGAAGATCAGAAGTGAGATCGAAGCAATAAAGAAGATTATAGCTGAGTTTGACGTCGTGAAGGAAAGTGTTAACGAACTCTCAGAAAAGGCTAAGACTGATCCACAAGCTGCAGAAAAACTTAATAAGCTAATTGAGGGATACACGTATGGTGAAGAACGAAAACTTTATGATAGCGCCTTGTCAAAAATTGAGAAATTGATTGAGACCTTATCCCCTGCCAGGTCTAAATCTCAGAGCACTATGAACCAGCGTAATAGGAATAATCGGAAAATTGTT |
| --- | --- |
| amino acid: | MSIVSQTRNKELLDKKIRSEIEAIKKIIAEFDVVKESVNELSEKAKTDPQAAEKLNKLIEGYTYGEERKLYDSALSKIEKLIETLSPARSKSQSTMNQRNRNNRKIV |

**CesD**

| nucleotide: | ATGAGTCGAAAGTTCAGCAGTTTGGAAGATATCTACGACTTCTACCAAGACGGAGGAACTCTAGCATCTTTAACTAATCTAACCCAACAGGATCTTAATGATCTGCATTCTTACGCATACACTGCATATCAATCCGGTGACGTAATTACAGCTAGAAACCTTTTTCATTTGTTAACCTACCTGGAACATTGGAACTACGATTATACACTCTCCTTAGGTTTGTGTCACCAGAGATTGTCAAACCACGAAGATGCTCAGCTTTGTTTTGCTAGGTGTGCAACACTTGTTATGCAAGATCCACGTGCTTCTTATTATTCAGGTATATCATATTTGCTCGTGGGCAACAAAAAGATGGCTAAAAAGGCCTTTAAAGCCTGCCTTATGTGGTGCAATGAAAAAGAGAAATATACTACATATAAGGAGAATATTAAGAAGCTGCTCGGGAATACTGAG |
| --- | --- |
| amino acid: | MSRKFSSLEDIYDFYQDGGTLASLTNLTQQDLNDLHSYAYTAYQSGDVITARNLFHLLTYLEHWNYDYTLSLGLCHQRLSNHEDAQLCFARCATLVMQDPRASYYSGISYLLVGNKKMAKKAFKACLMWCNEKEKYTTYKENIKKLLGNTE |

**CesD2**

| nucleotide: | ATGGTCGATACTTTCAACGACGAAGTATTCAACTACTACCTGGAACAAAAGGGTTACACAATCCAAAAGGAATTCCTGTGCGGATCAGCATTTTTCATCGGTTGGAGAATTGAAACACCTTTTTTCAGTCTAGCATATCGTCTCGATGAACAAGAGTTGATTCTCTGTAGTTTTGAGGCTAGAAACCAAACGGGTTTAAATGGACCAGTTCTTTCACTTACCCATCTTTTGGAGGAATTGTATCATCACTTTTCCGGAATAAAGAAGATTAGCGCTATGAAGAGCAAAATAGGCTCTGACTCTGAGCGACAGAAAAGGGAAGAGTTATTTAATTATTTTATTCGGAAAGGGGCCGTTCAGCAGGAGACTGAGGATGGGATTTGGTTTGTTATGAATGTGAATTCT |
| --- | --- |
| amino acid: | MVDTFNDEVFNYYLEQKGYTIQKEFLCGSAFFIGWRIETPFFSLAYRLDEQELILCSFEARNQTGLNGPVLSLTHLLEELYHHFSGIKKISAMKSKIGSDSERQKREELFNYFIRKGAVQQETEDGIWFVMNVNS |
