# Supplementary figures and images for: Co-expression with the Type 3 Secretion Chaperone CesT from Enterohemorrhagic E. coli Increases Accumulation of Recombinant Tir in Plant Chloroplasts
Source: Front Plant Sci. 2017 Mar 6;8:283. doi: 10.3389/fpls.2017.00283 (PMC5337511; doi:10.3389/fpls.2017.00283)

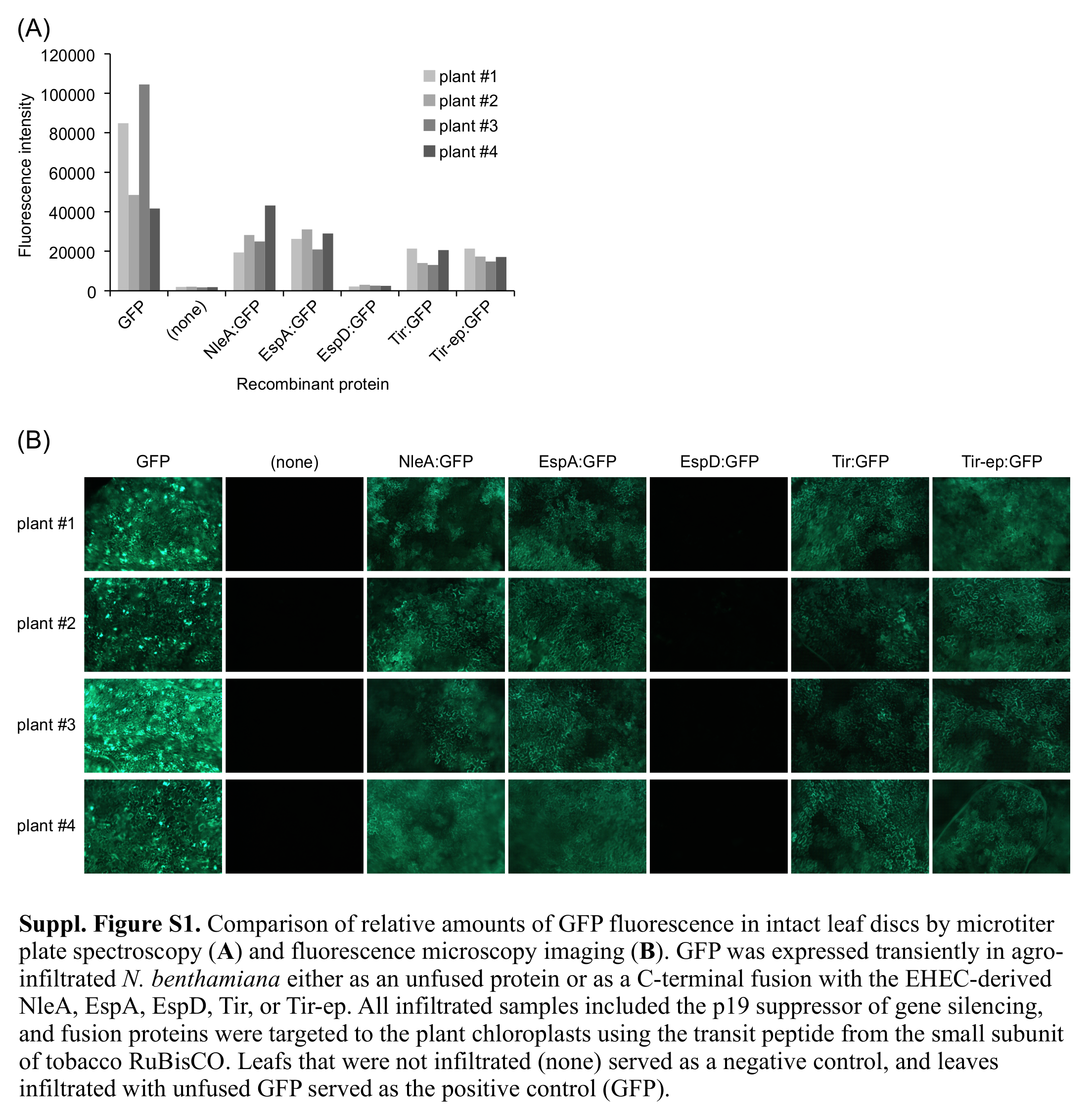

Supplement: Supplementary file 2 [file Image_1.TIF]
